# The epithelial sodium channel in inflammation and blood pressure modulation

**DOI:** 10.3389/fcvm.2023.1130148

**Published:** 2023-04-12

**Authors:** Taseer Ahmad, Lale A. Ertuglu, Sepiso K. Masenga, Thomas R. Kleyman, Annet Kirabo

**Affiliations:** ^1^ Department of Pharmacology, College of Pharmacy, University of Sargodha, Sargodha, Pakistan; ^2^ Division of Clinical Pharmacology, Department of Medicine, Vanderbilt University Medical Center, Nashville, TN, United States; ^3^ Division of Nephrology, Department of Medicine, Vanderbilt University Medical Center, Nashville, TN, United States; ^4^ Department of Physiological Sciences, School of Medicine and Health Sciences, Mulungushi University, Livingstone, Zambia; ^5^ Department of Medicine, University of Pittsburgh, Pittsburgh, PA, United States; ^6^ Department of Cell Biology, University of Pittsburgh, Pittsburgh, PA, United States; ^7^ Department of Pharmacology and Chemical Biology, University of Pittsburgh, Pittsburgh, PA, United States

**Keywords:** epithelial sodium channel (ENaC), δ-ENaC, antigen presenting cells, inflammation, endothelium dysfunction, blood pressure

## Abstract

A major regulator of blood pressure and volume homeostasis in the kidney is the epithelial sodium channel (ENaC). ENaC is composed of alpha(α)/beta(β)/gamma(γ) or delta(δ)/beta(β)/gamma(γ) subunits. The δ subunit is functional in the guinea pig, but not in routinely used experimental rodent models including rat or mouse, and thus remains the least understood of the four subunits. While the δ subunit is poorly expressed in the human kidney, we recently found that its gene variants are associated with blood pressure and kidney function. The δ subunit is expressed in the human vasculature where it may influence vascular function. Moreover, we recently found that the δ subunit is also expressed human antigen presenting cells (APCs). Our studies indicate that extracellular Na^+^ enters APCs *via* ENaC leading to inflammation and salt-induced hypertension. In this review, we highlight recent findings on the role of extra-renal ENaC in inflammation, vascular dysfunction, and blood pressure modulation. Targeting extra-renal ENaC may provide new drug therapies for salt-induced hypertension.

## Introduction

Hypertension affects more than 1 billion people around the globe. While our understanding of hypertension and the available treatments has improved vastly over the decades, blood pressure control remains a challenge in many hypertensive patients (1, 2). The etiology of hypertension is multifactorial, including environmental, genetic, and demographic factors (3). High Na^+^ intake is one of the key environmental risk factors for elevated blood pressure (4). However, salt sensitivity of blood pressure, which is a phenotype characterized by changes in blood pressure that correspond to dietary salt intake, is not uniform in humans (5). Many mechanisms have been described to explain the variability of salt sensitivity. These include genetic variations related to the renin-angiotensin-aldosterone system (RAAS), renal Na^+^ transporters, sympathetic nervous system and vascular dysfunction (6, 7). Recently, inflammation has been found to be a key modulator of salt sensitive blood pressure response (8). Indeed, inflammatory cytokines and reactive oxygen species (ROS) induce vascular endothelial dysfunction and impair renal Na^+^ excretion, resulting in blood pressure elevation (9, 10).

ENaC dependent reabsorption of Na^+^ in the aldosterone-sensitive distal nephron (ASDN) has a role in regulating extracellular fluid volume and blood pressure (11). The channel also facilitates K^+^ secretion in the ASDN (12). ENaC is thought to be composed of αβγ or δβγ subunits, encoded by genes *SCNN1A* (α subunit), *SCNN1B* (β subunit), *SCNN1G* (γ subunit), and *SCNN1D* (δ subunit) respectively, that are members of the ENaC/Degenerin superfamily (13). Other subunit stoichiometries have been reported in specific tissues. For example, the channel in mouse dendritic cells (DCs) has only α and γ subunits (14–16).

In the aldosterone-sensitive distal nephron of human kidney, ENaC is composed of the α, β, and γ, as the δ subunit is poorly expressed in this nephron segment (5). Besides its role in renal Na^+^ and K^+^ handling in the kidneys, ENaC affects blood pressure through its actions in various extrarenal tissues. ENaC in the lingual epithelium mediates salt taste and influences Na^+^ ingestion, while ENaC in the distal colon serves as the final site for absorption of ingested Na^+^. Neurons in the rostral ventral medulla of the brain sense increases in [Na^+^] in an ENaC dependent manner, leading to increase in sympathetic nerve activity and high blood pressure (17, 18). In animal models, such as the mouse and rat, ENaC has been proposed as a key protein for salt taste, but its contribution to human salt taste is less clear (19, 20). Recently, we found that extracellular Na^+^ enters APCs *via* ENaC leading to formation of lipid peroxidation products known as Isolevuglandins (IsoLGs), release of proinflammatory cytokines and salt-induced hypertension (14, 16, 21). Moreover, we found that the δ subunit is the most expressed subunit in human APCs (15). In a recent analysis of phenotypic and whole genome sequence data within the Trans-Omics in Precision Medicine project (TOPMED), we found that low frequency and rare variants of α, β and δ subunits of ENaC are associated with blood pressure, and β, δ subunits were associated with estimated glomerular filtration rate (22). Although the δ subunit is poorly expressed in the human kidney, it was the subunit where variants were associated with all blood pressure parameters analyzed, including pulse pressure, systolic, diastolic, and mean arterial pressure as well as kidney function (22). These studies indicate that extrarenal the δ subunit plays an important role in blood pressure regulation. In this review, we provide an overview of the latest findings relating to the roles of extrarenal ENaC, and its δ subunit in inflammation, vascular dysfunction, and blood pressure modulation (21, 23).

### Basic structure of ENaC

**Figure 1:**
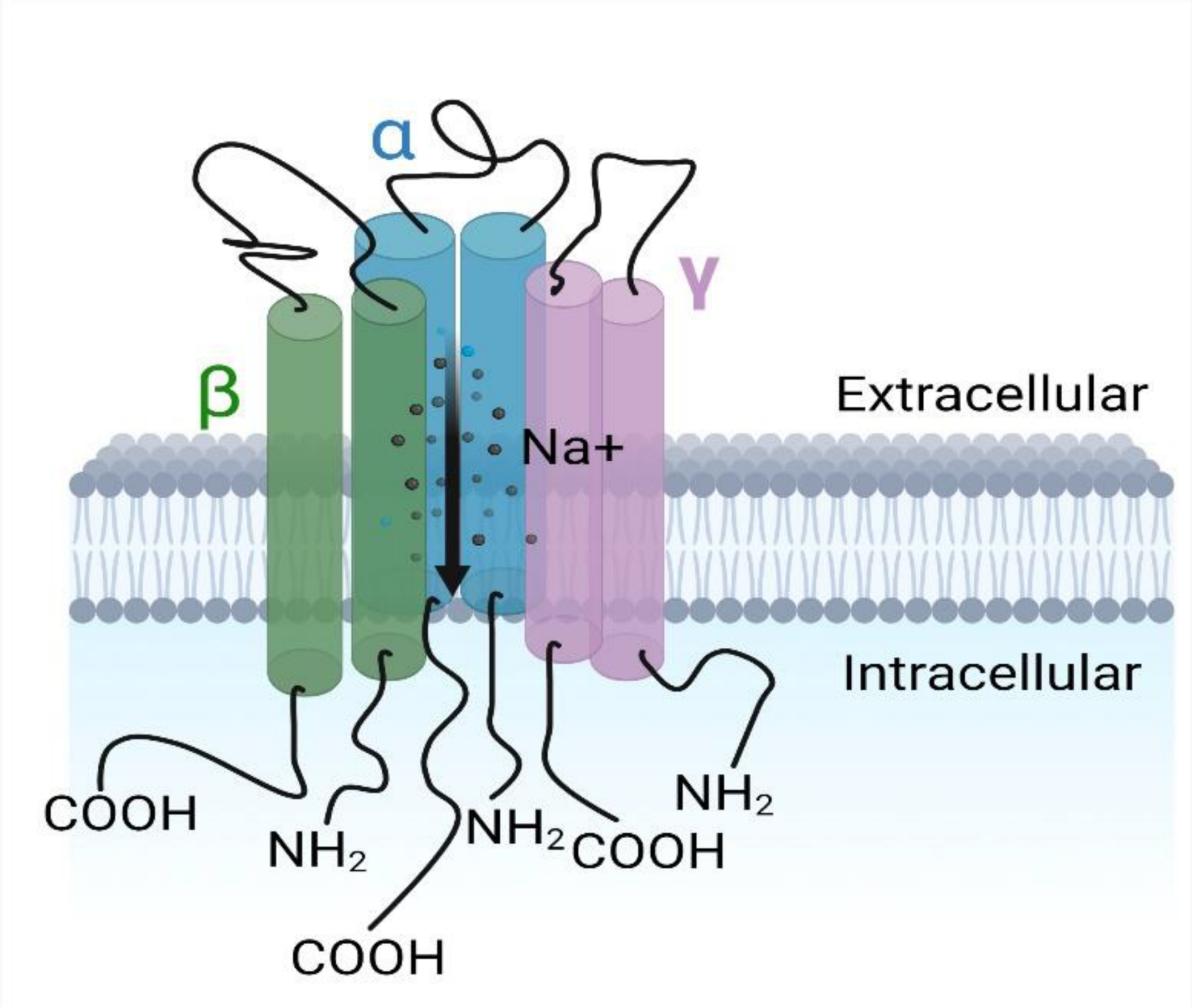
**The epithelial Na^+^ channel.** Each subunit (α, β, γ) having the carboxyl and amino termini groups intracellular with two (2) transmembrane segments each and a large extracellular domain between them.

Ion channels are found in all cells of the body serve to selectively transport ions such as Na^+^, K^+^ and Ca^2+^ across cell membranes (24). ENaC is an amiloride-sensitive, voltage-independent, trimeric constitutively-active ion channel formed by structurally related subunits with two transmembrane-spanning regions, intracellular COOH and NH_2_ termini, connected by a large extracellular domain/loop as shown in Figure 1 (24). The extracellular loop has a “hand -like” structure, consisting of a “palm”, “ball”, “finger”, “thumb”, “β-ball” and “knuckle” domain (25). These extracellular regions interact with various stimuli that modulate channel activity (26–28). The ENaC structure reveals that it assembles with a 1:1:1 stoichiometry of α, β, γ subunits arranged in a counter-clockwise manner (29).

Since its cloning in 1993 and 1994 by Canessa and colleagues (30), key features regarding ENaC's structure and regulation have been described in detail (31, 32). Expressed in the apical plasma membrane of epithelia, ENaC mediates the first step of transepithelial Na^+^ reabsorption in the ASDN of the kidney, airway and alveolar cells, distal colon and sweat ducts. It is involved in the regulation of blood pressure and extracellular [K^+^], and gene variants within genes encoding ENaC subunits (*SCNN1*A, *SCNN1*B, *SCNN1*G and *SCNN1*D) influence blood pressure (22, 33). ENaC also has crucial roles in the homeostasis of lung alveolar fluid (34, 35).

### The delta subunit of the epithelial sodium channel

ENaC was initially thought to consist of three subunits: α, β, γ (36). The fourth subunit, δ, was later identified (37, 38). While ENaC is formed by the combination of three homologous αβγ subunits in kidney (36, 39), the δ-subunit may replace α to form δβγ in other tissues (40). It is generally accepted that for proper channel function, ENaC must be composed of an α or δ subunit. Co-expression of β and γ subunits augment channel activity in heterologous expression systems, contribute to the channel pore and influence channel properties (38, 41). The α, β and γ subunits are primarily expressed in epithelial cells of the kidney and colon. While the δ subunit is expressed in lung, it is also expressed in non-epithelial tissues including, heart, brain, vasculature, and immune cells (38). All four subunits (αβγδ) of ENaC are also expressed in the normal human eye (42). Interestingly, in human lung epithelial cells the δ subunit may form multimeric channels with the β and γ subunits that could account for functional heterogeneity of the channel in this tissue (43). The biophysical and pharmacological properties of human αβγ and δβγ differ (44). Human αβγ channels are activated by proteases and inhibited by extracellular Na^+^, effects that are largely absent in δβγ channels. Furthermore, human δβγ channels have a higher single channel Na^+^ ion conductance and a higher amiloride IC_50_, compared to human αβγ channels (2, 45).

The δ subunit is expressed at sites, including specific locations in the brain, APCs and vasculature, where it likely has a role in Na^+^ sensing rather than transepithelial Na^+^ transport (38, 46). While αβγ ENaC has been extensively studied in mice and rats, studies of δ ENaC have lagged, in large part due to its expression as a pseudogene in these rodents albeit guinea pigs express functional δβγ channels (47). Furthermore, studies using humanized rodent models may provide a solution to understand the role of the δ subunit in blood pressure regulation.

## ENaC in the vasculature

Recent work suggests that δ subunit variants are associated with vascular function and blood pressure (22, 40, 47–49). ENaC in endothelial cells influences vascular tone by increasing intracellular Na^+^, stabilizing f-actin, and inhibiting endothelial nitric oxide synthase (eNOS), leading to endothelial stiffening, and reduced nitric oxide production (12, 50–52). This work has largely been performed in cultured cells. This regulatory pathway may be relevant *in vivo*, as mice with an endothelial γ subunit knockout have increased eNOS levels and eNOS activation (52). In addition, elevation of intracellular Na^+^ concentration hindered the transportation of l-arginine, resulting in impaired generation of nitric oxide (53, 54). However, the exact role of endothelial ENaC in the regulation of blood pressure is still unclear. Elevated expression and increased activity of ENaC can result in vascular dysfunction in some rodent animal models (40). While there is limited knowledge on the expression and function of ENaC channels in human vasculature, the δ-subunit has been reported to be expressed in human endothelial cells, where functional αβγ and δβγ channels are been observed at the single channel level (40, 55). Vascular endothelium is a target for aldosterone, where it regulates ENaC expression in a mineralocorticoid-dependent manner (50). ENaC expression affects endothelial cell stiffness and nitric oxide synthesis in cultured human endothelial cells (56).

**Figure 2:**
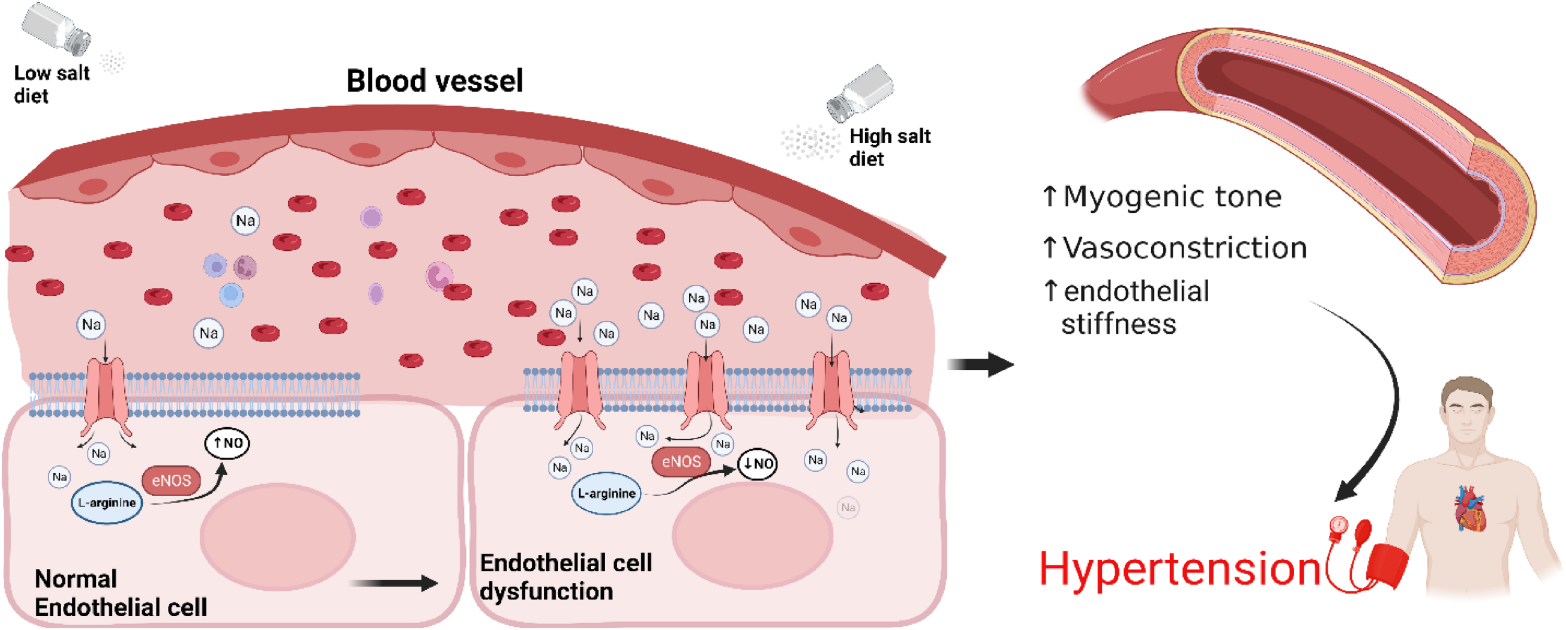
**Sodium induces an ENaC-dependent transition from endothelial function to dysfunction**. High salt diet leads to overexpression of ENaC and reduced nitric oxide production, increased myogenic tone, vasoconstriction, arterial stiffness resulting in hypertension. Endothelial cells at the bottom have been enlarged for illustration purposes. NO, nitric oxide; eNOS, endothelial nitric oxide synthase.

*In vitro* studies in human endothelial cell lines suggested that elevated levels of extracellular Na^+^ results in enhanced Na^+^ entry into endothelial cells *via* ENaC, increasing endothelial stiffness and reducing nitric oxide generation, potentially altering vascular tone (Figure 2) (50). However, to date there are no publications demonstrating that a knockout of ENaC subunits in endothelial cells affects blood pressure. Recently it was reported that functional ENaC subunits are expressed in the human aorta and internal mammary artery and their expression levels are associated with hypertension (48, 50). Reduced expression of the *γ* subunit was observed in the aorta of hypertensives with controlled blood pressure compared to aorta from normotensive individuals, while reduced expression of δ subunit was observed in internal mammary arteries from controlled hypertensives compared to normotensive individuals (40, 57). While interesting, the observations are correlative, based on small numbers and need to be confirmed.

## Renal ENaC and hypertension

ENaC gain- and loss-of-function mutations have profound effects on blood pressure in individuals with Liddle syndrome and pseudohypoaldosteronism type I, respectively (23, 58–63). Mutations that result in activation of αβγ channels cause Liddle syndrome, a hereditary form of hypertension (64). In contrast, ENaC loss of function mutations are observed in pseudohypoaldosteronism type 1 and cause salt-wasting and hypotension. Based on these observations, factors that increase ENaC activity in the kidney have been suggested to contribute to the genesis of salt-sensitive hypertension (63, 65–67). Aldosterone is a major regulator of ENaC activity through the mineralocorticoid receptor, which tightly controls renal Na^+^ absorption and K^+^ secretion in the ASDN, thus regulation both the extracellular fluid volume (ECFV) and serum [K^+^]. Primary hyperaldosteronism, which is due to poorly regulated aldosterone secretion in the adrenal gland, is a well-known cause of secondary hypertension. It is estimated that about 10% of hypertensives have primary hyperaldosteronism (68, 69) and this number increases to 20% in drug-resistant hypertensives (64, 70). As approximately 90% of the US adult population consumes significantly more salt than the daily recommendation of <2,300 mg (71), the vast majority of patients with primary hyperaldosteronism are estimated to have high salt intake (72).

Aldosterone also has been associated with proinflammatory immune effects. Dietary salt and aldosterone also have synergistic effects on the expression of proinflammatory cytokines (73, 74). In addition to mineralocorticoid antagonists, pharmacological blockade of ENaC with amiloride is an important therapeutic option for patients with refractory hypertension and primary hyperaldosteronism (75, 76). Interestingly, ENaC inhibition by amiloride is effective in reducing blood pressure in patients with hypertension resistant to mineralocorticoid antagonism (77). This phenomenon may be explained by ENaC's expression in the vascular smooth muscle of renal, mesenteric and cerebral arteries (8, 78, 79) where its inhibition may result into the loss of myogenic response and pressure-induced vasoconstriction (49). Emerging evidence also suggest that systemic ENaC inhibition may modulate salt-induced immune activation and inflammation-mediated end-organ damage. However, its unclear whether amiloride achieves sufficient levels in the systemic circulation to affect non-renal ENaCs in humans.

## Extra-renal ENaC in inflammation and salt-sensitive hypertension

While the role of ENaC in the pathogenesis of salt-sensitive hypertension through renal volume and Na^+^ homeostasis has been extensively studied, emerging evidence points to a role of extra-renal ENaC in blood pressure regulation through inflammatory pathways. Immune activation and inflammation play a well-established role in hypertension. Both innate and adaptive immunity are fundamental in the development of hypertensive responses to salt and related vascular and renal dysfunction, as previously reviewed elsewhere (80, 81). Pro-hypertensive stimuli first activate APCs, including DCs and macrophages, which, in turn, activate T-cells through antigen-MHC receptor interaction (82). Inhibition of this interaction abolishes deoxycorticosterone acetate (DOCA)-salt induced hypertension (83). High dietary salt intake results in infiltration of APCs and T-lymphocytes into the kidneys that cause vascular remodeling, renal Na^+^ retention and subsequent hypertension (8, 84–86).

Importantly, recent evidence suggests that ENaC plays a critical role in the association between inflammation and salt-sensitive hypertension. Antigen-presenting DCs and monocytes in humans express all ENaC subunits, with the δ subunit exhibiting highest expression levels (21). In contrast, the mouse splenic DCs express the α and γ subunits of ENaC, but β subunit is absent (12, 16).

**Figure 3:**
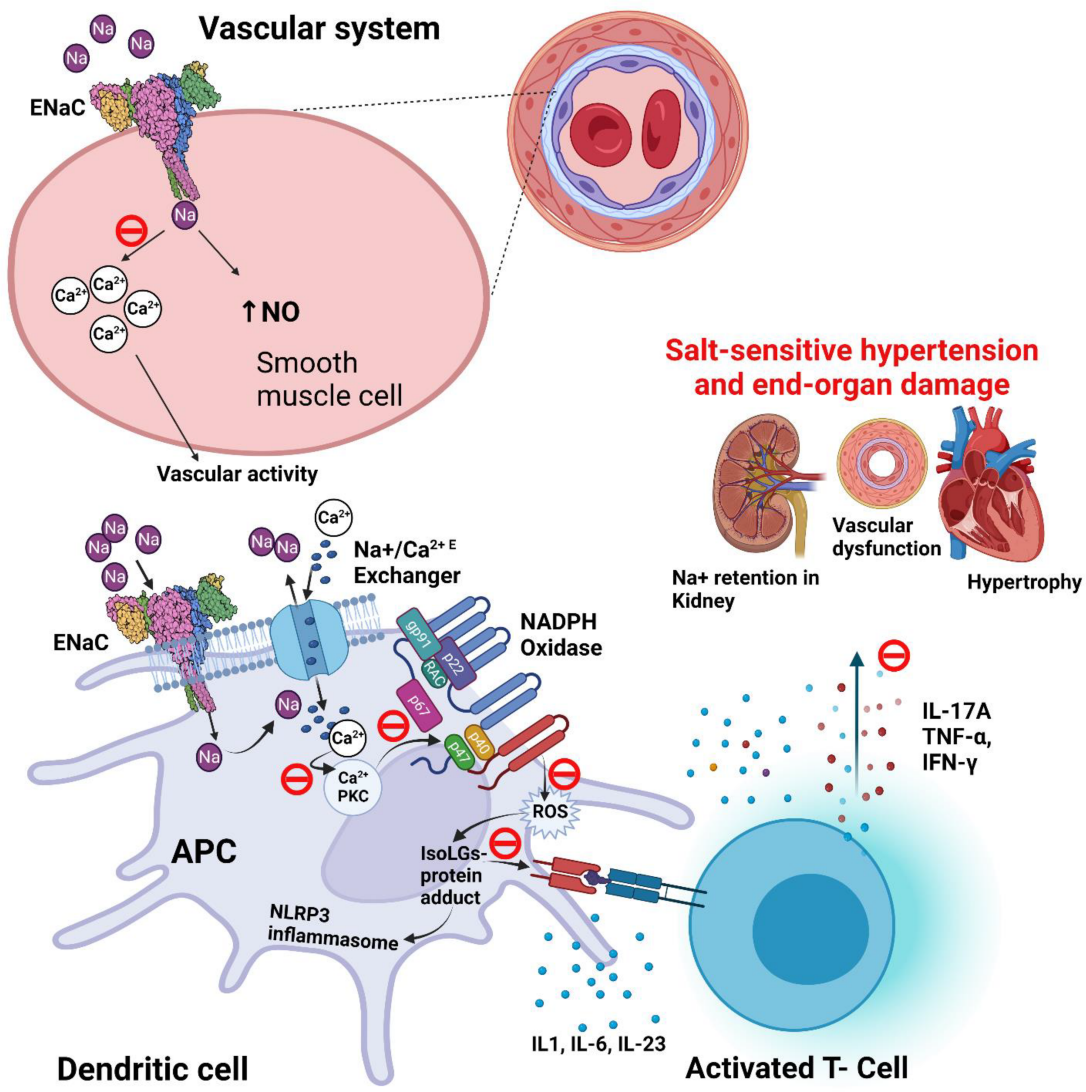
**Potential role and proposed blockade mechanisms of ENaC in salt-sensitive hypertension.** ENaC (epithelial sodium channel) and IsoLG (isolevuglandins) dependent activation of NLRP3 [NOD (nucleotide-binding and oligomerization domain)-like receptor family pyrin domain containing 3] inflammasome in salt-sensitive hypertension. Also shows the proposed blockade of signaling pathway of ENaC (θ; blockade icon), and epithelial sodium channel (ENaC) role in the regulation of blood pressure in smooth muscle of blood vessel. APC, antigen presenting cell; IsoLG, Isolevuglandins; NO, nitric oxide; NADPH, nicotinamide adenine dinucleotide phosphate; P, phosphorylation; PKC, protein kinase C; ROS, reactive oxygen species.

We previously found a pathway by which ENaC-mediated Na^+^ entry into APCs leads to T-cell activation and subsequent elevation in blood pressure (87). Elevated extracellular Na^+^ enters APCs *via* ENaC (88). Once inside the cell, Na^+^ is exchanged with Ca^2+^, leading to an increase in intracellular Ca^2+^ and activation of protein kinase C (PKC). PKC phosphorylates and activates NADPH oxidase and leads to the formation of isolevuglandins (IsoLGs; also called Isoketals or γ-ketoaldehydes). IsoLGs are highly reactive oxidative products of arachidonic acid metabolism and adduct to proteins through the lysine residues. The resulting IsoLG-protein adducts are highly immunogenic and are presented on the MHC-II cell surface receptors that activates T cells. This salt-induced, ENaC-mediated immune cell activation leads to the secretion of pro-inflammatory cytokines including IL-1β and IL-6 from the APCs and IFN-γ and IL-17A from the T cells. Tissue infiltration by these cells and the release of inflammatory cytokines results in vascular and kidney dysfunction leading to hypertension (Figure 3) (85). Recent evidence indicates that ENaC-mediated Na^+^ entry also triggers the NOD [nucleotide-binding and oligomerization domain]-like receptor family pyrin domain containing 3 (NLRP3) inflammasome activation in APCs, another important instigator of hypertensive response (15).

Inflammasomes are intracellular sensors of pathogen-associated molecular patterns (PAMPs) and endogenous host-derived damage-associated molecular patterns (DAMPs). The NLRP3 inflammasome can be activated through the canonical and non-canonical pathways and results in the release of pro-inflammatory cytokines IL-1β and IL-18, as well as gasdermin D induced pyroptotic cell death (89). The canonical pathway includes a priming and an activation signal. The priming signal, which is provided by Toll-like receptors (TLRs), the nucleotide-binding oligomerization domain (NOD) 1 and 2 or cytokine receptors, activates the nuclear factor kappa B (NF-*κ*B), which results in the expression of NLRP3 and pro-IL-1β (90). After priming, various signals including pathogen components, microbial toxins, and cellular signals such as ion influx, reactive oxygen species (ROS), mitochondrial and lysosomal damage, can serve as the second signal for activation of NLRP3 (91). Patients with hypertension are characterized by high plasma levels of IL-1β and IL-18, the main end-products of NLRP3 inflammasome activation (92–94), which also associates with end-organ damage (95).

Further evidence suggesting a link between NLRP3 inflammasome and hypertension has been provided by genetic studies showing an association between high blood pressure and single nucleotide polymorphism in NLRP3 gene, rs7512998 (96) as well as tandem repeat polymorphism in NLRP3 gene CIAS1 (97, 98).

In animal models of salt sensitive hypertension, absence, or inhibition of NLRP3 inflammasome has been found to prevent the development of hypertension and associated renal damage (99, 100). Similarly, NLRP3 inflammasome, IL-1 receptor and inflammasome-mediated immune cell activation were shown to be essential in the development of aldosterone-induced vascular damage (101). Inhibition of NF-*κ*B, an essential component of canonical pathway for inflammasome activation, induces vasodilation (102), decreases blood pressure and protects against hypertensive end-organ damage (103, 104).

Recent studies in our laboratory have also shown a crucial role of NLRP3 inflammasome in ENaC- and IsoLG-dependent APCs activation and subsequent inflammation in salt-sensitive hypertension (105). Using cell hashing, and cellular indexing of transcriptomes and epitopes of peripheral blood mononuclear cells, we found that NLRP3 inflammasome expression and IL-1β echo changes in blood pressure induced by salt depletion. *In vitro* exposure of human monocytes to high Na^+^ increases the expression of caspase-1, IL-1β and IL-18. In further studies using mouse models of salt-sensitive hypertension, we found that APCs from salt-sensitive mice have higher salt-induced intracellular IL-1β production. Following 4-weeks of high salt diet, DCs and monocytes exhibited increased accumulation of NLRP3, IL-1β along with IsoLG-protein adducts. Pharmacological inhibition of NLRP3 inflammasome attenuates salt-induced increase in APC NLRP3, IL-1β and IsoLG-adducts as well as salt-sensitive blood pressure elevation. These findings show that ENaC mediated Na^+^ entry in the APCs induce immune cell activation and inflammation through IsoLG-formation, NLRP3 inflammasome activation and release of pro-inflammatory cytokines that orchestrate vascular and renal dysfunction and ensuing salt-sensitive hypertension (21, 85). These new insights imply that ENaC may play a pivotal role in the regulation of blood pressure through its actions in immune cells in addition to its well-recognized function in the nephron (106). So, the recent pivotal discoveries related to the presence and functioning of extra-renal ENaC in immune cells may illuminate additional therapeutic targets for ENaC in salt-induced blood pressure (8).

## Conclusion

Recent evidence from human and animal studies has expanded our understanding of ENaC and its subunit δ in relation to its functional roles in non-epithelial tissues and blood pressure regulation. It is now well-recognized that the immune system plays an active role in the development and progression of hypertension, and high salt intake not only drives hemodynamic changes but is also associated with inflammation. Thus, inhibition of extra-renal ENaC, including the δ subunit, or an intermediate step in this signaling pathway (as shown in Figure 3), may improve blood pressure control in patients with salt-sensitive hypertension (107). A major side effect of ENaC inhibitors is hyperkalemia (108). The development of drugs that selectively target δβγ channels, while not blocking αβγ channels in the human kidney should prevent drug-induced hyperkalemia. Furthermore, future studies should investigate potential novel drugs specifically targeting APCs, ENaC, NLRP3, PKC or oxidative stress for safety and efficacy (as shown in Figure 3). Lastly, as high salt intake is consistently associated with a rise in blood pressure (109) and has a more pronounced influence on salt-sensitive populations, reductions in dietary salt could provide a cost effective approach to decrease the risk of hypertension and related cardiovascular disease (110).
